# Monkeypox DNA levels correlate with virus infectivity in clinical samples, Israel, 2022

**DOI:** 10.2807/1560-7917.ES.2022.27.35.2200636

**Published:** 2022-09-01

**Authors:** Nir Paran, Yfat Yahalom-Ronen, Ohad Shifman, Shirley Lazar, Ronen Ben-Ami, Michal Yakubovsky, Itzchak Levy, Anat Wieder-Feinsod, Sharon Amit, Michal Katzir, Noga Carmi-Oren, Ariela Levcovich, Mirit Hershman-Sarafov, Alona Paz, Rebecca Thomas, Hadas Tamir, Lilach Cherry-Mimran, Noam Erez, Sharon Melamed, Moria Barlev-Gross, Shay Karmi, Boaz Politi, Hagit Achdout, Shay Weiss, Haim Levy, Ofir Schuster, Adi Beth-Din, Tomer Israely

**Affiliations:** 1 Department of Infectious Diseases, Israel Institute for Biological Research, Ness-Ziona, Israel; 2 Department of Biochemistry and Molecular Genetics, Israel Institute for Biological Research, Ness-Ziona, Israel; 3 Infectious Diseases Department, Tel Aviv Sourasky Medical Center, Tel Aviv, Israel; 4 Infectious Disease Unit, Sheba Medical Center, Ramat Gan, Israel; 5 Clinical Microbiology, Sheba Medical Center, Ramat Gan, Israel.; 6 Infectious Disease unit, Meir Medical Center, Kfar Saba, Israel; 7 Infectious Disease unit, Shamir (Assaf Harofeh) Medical Center, Be'er Ya'akov, Israel; 8 Infectious Disease and Control Unit, Bnai Zion Medical Center, Haifa, Israel; 9 Department of Emergency Medicine, Rabin Medical Center, Beilinson Hospital, Petah Tikva, Israel; 10 Sackler medical school, Tel Aviv University, Tel Aviv, Israel; 11 Rappaport Faculty of Medicine, Technion-Israel Institute of Technology, Haifa, Israel

**Keywords:** Monkeypox, PCR, Cq, pfu, threshold, infectivity

## Abstract

The current monkeypox virus global spread and lack of data regarding clinical specimens’ infectivity call for examining virus infectivity, and whether this correlates with results from PCR, the available diagnostic tool. We show strong correlation between viral DNA amount in clinical specimens and virus infectivity toward BSC-1 cell line. Moreover, we define a PCR threshold value (Cq ≥ 35, ≤ 4,300 DNA copies/mL), corresponding to negative viral cultures, which may assist risk-assessment and decision-making regarding protective-measures and guidelines for patients with monkeypox.

In early May 2022, monkeypox virus (MPXV) human infections were reported in the United Kingdom (UK). Since, increasing numbers of cases, also attributed to the MPXV Clade II, have been observed in other non-endemic countries for the virus, amounting, up to 5 August 2022, to over 28,220 cases worldwide [1 ]. To date, the current outbreak is mostly affecting men who have sex with men (MSM), and the disease caused by MPXV is manifested mainly with skin/mucosal lesions at multiple sites including genital, oropharyngeal or perianal areas [2]. Presently, the rapid detection and identification of MPXV relies on a PCR from a patient (or an environmental) sample being positive for MPXV genetic material (DNA). However, the presence of viral DNA in a clinical specimen does not imply its infectivity, restricting risk assessment [3]. In this study, we test for a correlation between MPXV DNA copies in patient specimens, as measured by PCR, and infectious virus, as measured by plaque assay. We also estimate a threshold value in PCR that may inform decision-making on MPXV infection control.

## Determination of viral DNA copies in clinical samples and infectivity of the virus from each sample in cell cultures

A total of 43 clinical specimens from 32 male patients from six medical centres in Israel were included in this study. The specimens consisted of 21 oropharyngeal swabs, 20 dermal-lesion exudate swabs, and two rectal swabs (Supplemental Table S1). Among the 32 patients, 10 had paired oropharynx and dermal-lesion exudate swabs and one patient had both oropharyngeal and rectal swabs (Supplemental Table S1). Swabs were stored in viral-transport-medium-(VTM) containing tubes up to 48 hours at 4°C before being subjected to viral DNA quantification (via the quantitation cycle (Cq) value in PCR) or infectious virus titre measurement (pfu/mL in plaque assay).

To determine the Cq value for each specimen, MPXV DNA was extracted with the QIAamp DNA Mini Kit (Qiagen) using a protocol for blood and body fluids available from the manufacturer, in a QIAcube robot, and multiplex real-time PCR assays were performed using MPXV generic assay primers, as well as primers specific for MPXV Clade II (Supplemental Material). Cq values, one for each of these paired PCRs conducted on the respective 43 specimens were determined, the lowest of which was retained as the specimen Cq.

In addition, for standardisation, DNA concentrations (copies/mL) were derived from the Cq values using a standard curve based on calibrated MPXV DNA standard.

To determine the MPXV infectivity of the same 43 samples, a plaque assay was employed. The VTM solutions, where swabs had respectively been stored, each underwent 10-fold serial dilutions, and the latter were used to infect BSC-1 cells (Supplemental Material). Plates were fixed and stained 72 hours later, plaques were counted, and plaque forming units/mL (pfu/mL) were determined.

## Correlation between viral DNA quantity in specimens and virus infectivity in cell culture

**Figure 1:**
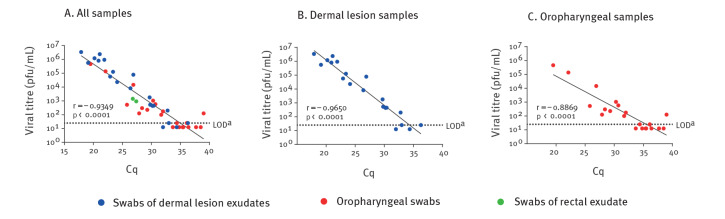
Correlation between quantity of viral DNA and infectious virus titre in clinical specimens of MPXV infected patients, Israel, 2022 (n = 43 specimens)

Next, we aimed to determine the correlation between quantity of viral DNA in each sample and infectious virus titre. Correlation and its significance were determined by Pearson correlation test. By plotting Cq values against log (pfu/mL) of all samples irrespective of swab type, a significant negative correlation was found (Figure 1A, r = ‒ 0.9349, p < 0.0001), with low Cq values correlating with high log (pfu/mL). Thus, high Cq values, representing a specimen with low viral DNA amount, indicate lower infectivity towards BSC-1 cells. Of note, most lesion swabs yield low Cq values and high viral load, whereas most oropharyngeal specimens display the opposite.

Cq: quantitation cycle; LOD: limit of detection (25 pfu/mL); MPXV: monkeypox virus; pfu: plaque forming units.

^a^ LOD = 25 pfu/mL.

Forty-three clinical samples (21 oropharyngeal swabs, 20 dermal lesion exudate swabs and two rectal swabs) from 32 patients were each subjected to MPXV-DNA quantification by real-time PCR and infectious-viral-titre determination by plaque assay. PCR results (Cq values), and corresponding infectious virus titres (pfu/mL) are plotted, with samples having a titre below LOD being assigned a value of half the LOD (12.5 pfu/mL). Plots for (A) all samples, (B) dermal lesion exudate samples only, and (C) oropharyngeal samples only are respectively presented with the Pearson correlation coefficient obtained.

To examine whether PCR values of oropharyngeal swabs correlate with infectivity similarly to dermal-lesion-exudate swabs, we correlated Cq values and log pfu/mL values for each specimen source separately. A significant correlation for both dermal lesions (r = ‒ 0.9650) and oropharynx swabs (r = ‒ 0.8869) was found (Figure 1B,C). Thus, Cq values in our study conditions strongly predict infectivity in cell lines for both specimen types.

**Figure 2:**
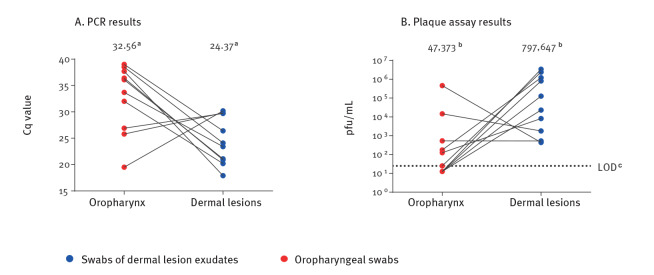
(A) Quantities of viral DNA and (B) infectious virus titres in paired oropharynx and dermal-lesion specimens from the same MPXV-infected patients, Israel, 2022 (n = 20 specimens)

Of the 43 specimens, 20 were paired swabs of oropharynx and dermal lesion from the same patient, thus enabling examination of the Cqs (Figure 2A) or pfu/mL (Figure 2B) per patient for these two specimen types. Most (7/10) tested patients appeared to present relatively higher viral loads (and lower Cq values) in dermal lesion specimens than in oropharynx specimens.

Cq: quantitation cycle; LOD: limit of detection (25 pfu/mL); MPXV: monkeypox virus; pfu: plaque forming units.

Paired samples (from oropharynx and dermal lesions) respectively derived from 10 individuals were tested by (A) PCR and (B) plaque assay. Results are plotted as dots, whose colour represent the sample type. Lines between different-coloured dots indicate two types of samples from the same patient.

^a^ Mean Cq value for the sample type.

^b^ Mean viral titre for the sample type.

^c^ LOD = 25 pfu/mL. Samples having a titre below LOD are assigned a value of half the LOD (12.5 pfu/mL).

## Determination of PCR threshold predicting non-infectious virus in cell cultures

Cq values for 10 of the tested oropharynx swabs were relatively high (> 33, Figure 1C). For most of them (7/10) infectious MPXV titres were below the limit of detection (LOD = 25 pfu/mL), two were at the LOD, and only one showed marginal viral load. Similarly, dermal lesions (Figure 1B) with Cq values greater than 33 (n = 3) were at or below LOD. To predict a Cq value that correlates with non-infectious virus, we calculated from the linear regression equation of all samples the Cq value corresponding to the LOD. The calculated Cq value was 34.98 (Figure 1A). 

This suggests that in clinical swabs, Cq values ≥ 35 predict no or very low infectivity. 

In addition, we calculated a ratio of 172 DNA copies for 1 pfu to provide a comparable value across different laboratories. Altogether, based on our limit of detection (25 pfu/mL), the minimum amount of DNA required for a clinical sample to be infectious is 4,300 DNA copies/mL.

## Discussion

MPXV belongs to the Orthopoxvirus genus within the Poxviridae family and is a double-stranded-DNA zoonotic virus [4,5]. Accordingly, MPXV transmission to humans is possible through interaction with wild animals and infectious fomites, however, close contact with infected people additionally represents a potential source of exposure [6].

MPXV was originally reported in humans in central Africa in 1970, and thereafter sporadic outbreaks were observed mostly in Central and West Africa, where the virus is endemic. Since 2003, sporadic MPXV outbreaks have also occurred in non-African countries [7]. Some of these were caused by importation of the virus through travel, such as in 2018, when MPXV belonging to Clade II was isolated in Israel, the UK and Singapore [7,8].

Since early May 2022, when a set of cases of MPXV were diagnosed in the UK, a monkeypox outbreak is spreading rapidly, currently spanning several countries, where the virus is not endemic. MPXV cases are being diagnosed by PCR testing that determines viral DNA presence in patients’ specimens. However, data on infectivity relative to viral DNA content are lacking, thus impairing risk assessment and determination of the appropriate protective measures [9]. In this investigation, we correlated PCR results from MPXV oropharyngeal, dermal lesion, and rectal swabs, with virus infectivity on cultured cells and found a strong negative correlation, predicting higher infectivity in specimens with low Cq values. 

We also tested paired swabs of oropharynx and dermal lesion from the same patients. Most tested patients appeared to present higher viral loads (and lower Cq values) in dermal lesion specimens than in oropharynx specimens, suggesting a higher infectivity risk from dermal lesions.

Based on our data, a Cq value of ≥ 35 predicts no or very low infectivity. This concurs with results of Lapa et. al., who reported the successful recovery of MPXV from semen, in a sample with a Cq value of 29 [10].

We further calculated a ratio of 172 DNA copies for 1 pfu. Previous reports suggest values of 10–100 DNA copies for 1 pfu for orthopox viruses originating from cultured cells [11,12]. This difference could be due to the presence of viral DNA not associated with infectious particles in our samples, or because viruses originating from clinical specimens might have a reduced infectivity towards cell lines, compared to viruses originating from culture. Keeping specimens refrigerated up to 48 hours prior to analysis might have also had an effect on our results. However, based on prior unpublished work in our laboratory (Supplementary Figure S2), poxvirus infectivity seems to be maintained following 24 hours of incubation at 4 °C and marginally affected upon incubation for 48 hours at the same temperature (up to 1.5-fold reduction, Supplementary Figure S2). Recent preliminary results with clinical specimens from the current MPXV outbreak additionally suggest that the virus infectivity is not affected after up to 48 hours of incubation at 4 °C (data not shown). This is in line with previous studies showing that poxviruses maintain their infection capacity for a relatively long time and across different conditions [13]. Furthermore, viruses derived from patients and/or the environment are commonly more resistant than virus material derived from cell cultures [13].

Overall, we suggest that a Cq value of ≥ 35 (≤ 4,300 DNA copies/mL) corresponds with non or marginal infectivity. Altogether, infectiousness should also be evaluated in context of the overall clinical manifestation including the course of disease, lesion location and stage, etc.

There are several limitations to this study. First, data on virus infectivity from other poorly available types of clinical specimens, such as semen, scabs, and rectal swabs still require further evaluation. Second, due to the nature of the disease that manifests mostly with dermal lesions and less with oropharyngeal ones [9], tested oropharyngeal swabs usually display relatively high Cq values, thus conclusions as to lower Cq values for oropharyngeal samples are limited. Analysis of additional lesion swabs with high Cq values would also be valuable. Furthermore, concerning the study specimens, data on the patients’ age range and whether they had comorbidities or received medication are not available. The time between sample collection and the estimated date of infection is not known either. It could also be that variation in specimen collection might have affected viral or DNA recovery. Nevertheless, the observed strong correlation between PCR and plaque assay results (Figure 1) derived from specimens collected in six medical centres, suggests that these factors are of minor effect.

## Conclusions

This work highlights the strong correlation of MPXV Cq values to virus infectivity and defines a threshold (Cq ≥ 35; viral DNA ≤ 4,300 copies/mL) that predicts poorly- or non-infectious specimens. These data, together with the corresponding clinical manifestation, may provide valuable support for decision-making regarding protective measures and guidelines for monkeypox patients and their close contacts.

## Supplementary Data

853000 Supplementary Material
