# Supplementary material for: Monkeypox DNA levels correlate with virus infectivity in clinical samples, Israel, 2022
Source: Euro Surveill. 2022 Sep 1;27(35):2200636. doi: 10.2807/1560-7917.ES.2022.27.35.2200636 (PMC9438394; doi:10.2807/1560-7917.ES.2022.27.35.2200636)
Supplement: Supplementary Material [file 2200636_SupplementaryMaterial.pdf]

Supplementary Material

This supplementary material is hosted by *Eurosurveillance* as supporting information alongside the article "Monkeypox DNA levels correlate with virus infectivity in clinical samples, Israel, 2022", on behalf of the authors who remain responsible for the accuracy and appropriateness of the content. The same standards for ethics, copyright, attributions and permissions as for the article apply. Supplements are not edited by Eurosurveillance and the journal is not responsible for the maintenance of any links or email addresses provided therein

Methods

Viruses and Clinical specimens:

Forty-three clinical specimens of oropharyngeal swabs (21/43), lesion exudate swabs (20/43), or rectal swabs (2/43) from male patients were tested. Samples were obtained from 32 patients. Ten out of the 32 patients have paired oropharyngeal-lesion exudate swabs. One patient has both oropharyngeal and rectal swabs (Table S1). Sample collection was performed by the medical centres.
Swabs were stored in viral-transport-medium (VTM) containing tubes up to 48h at 4°C. Tubes were vortexed for 1 minute before sample collection to either DNA extraction or plaque assay. PCR and pfu assays were performed at the IIBR.

Vaccinia virus (Lister) was propagated on Vero cells, purified by ultracentrifugation on 30% sucrose cushion and titrated by plaque assay as described [1].

DNA extraction:

DNA was extracted from 0.1 ml of the samples using the QIAamp DNA Mini Kit (Qiagen) using a protocol for blood and body fluids in a QIAcube robot and was eluted in 100 µl H_2O.

MPXV real time PCR:

Multiplex real-time PCR assays were adopted from Li et al [Supplementary ref 1] and performed in a 50 µl reaction volume using the SensiFAST™ Probe Lo-ROX kit (BIOLINE). The reaction mix contained 5 µl viral DNA sample, viral specific primers (30 pmol per reaction each) and probes (15 pmol per reaction each) detailed below: MPXV generic assay (GE):
forward primer (5′-GGAAAATGTAAAGACAACGAATACAG)
reverse primer (5′-GCTATCACATAATCTGGAAGCGTA)
probe (5′Joe-AAGCCGTAATCTATGTTGTCTATCGTGTCC-3′BHQ1)
MPXV Clade II specific assay (WA):
forward primer (5′-CACACCGTCTCTTCCACAGA)

reverse primer (5′-GATACAGGTTAATTTCCACATCG)

probe (5′FAM-AACCCGTCGTAACCAGCAATACATTT-3′BHQ1)

The PCR was carried out on a QuantStudio 5 real-time PCR system (Applied Biosystems), under the following conditions: 20 sec at 95°C followed by 40 cycles at 1 sec 95°C and 20 sec 60°C [2]. Cq values were extracted using automatic baseline determination and threshold of 0.1.

Calculation of MPXV DNA concentration:

Absolute DNA quantification (copies/reaction) was performed by Poisson-distribution-based qPCR approach, as described by Rosssmanith *et al*. [3]. Concentration was calculated from the number of positives and negatives of 96 qPCR parallel reactions each one containing 0.5× LOD DNA template.

A 10-fold dilution series of the calibrated sample, was used to create a standard curve.

Cells and Plaque assay:

BSC-1 (CCL-26, ATCC) and Vero (CCL-81, ATCC) cells were maintained in DMEM medium containing 10% Foetal Bovine Serum (FBS), MEM nonessential amino acids (NEAA), 2 mM L-glutamine, 100 Units/ml penicillin, 0.1 mg/ml streptomycin, 12.5 Units/ml nystatin (P/S/N), all from Biological Industries, Israel. For plaque assay, tenfold serial dilutions (-1 to -6) from each specimen starting from 1:10 dilution of the original clinical sample were performed in MEM medium containing 2% FBS, MEM nonessential amino acids (NEAA), 2 mM L-glutamine, 100 Units/ml penicillin, 0.1 mg/ml streptomycin, 12.5 Units/ml nystatin, all from Biological Industries, Israel. Monolayers of BSC-1 cells in 12-well plates were infected (200µl/well) in duplicates, incubated for 1 h at 37°C with 5% CO2 incubator. Then 2ml of methylcellulose overlay (MEM containing 0.5% methylcellulose, 2% FBS, 2 mM L-glutamine, 100 Units/ml penicillin, 0.1 mg/ml streptomycin, 12.5 Units/ml nystatin) were added to each well and cells were incubated for 72 h at 37°C with 5% CO2 incubator. Plates were fixed and stained with crystal violet, plaques were counted (Fig. S1). Plaques forming units (pfu) per ml was calculated based on number of plaques X 5 (to reach 1ml based on volume of 200µl/well) X dilution factor. LOD is based on the detection of 1 plaque per 1 out of 2 duplicate wells (=0.5) each infected with 200µl, for 1:10 dilution, hence: 0.5 X 5 (to reach 1ml based on volume of 200µl/well) X 10 (1:10) dilution factor = 25.

Handling and working with MPXV samples were conducted in a BSL3 facility in accordance with the biosafety guidelines of the Israel Institute for Biological Research (IIBR).

Statistics:

Statistical analyses were performed using GraphPad Prism 6.0. Linear regression was performed on log pfu/ml and Cq values. To measure the Correlation between Cq and pfu/ml values, we determined the Pearson correlation coefficient (r) for all samples as well as separately for dermal exudate and oropharyngeal swabs. Significance is presented as two-tailed p-value. Mean values are presented for Cq and pfu/ml.

patient #	oropharyngeal swabs		swabs from lesion exudate		rectal swabs	
	Cq value	pfu/ml	Cq value	pfu/ml	Cq value	pfu/ml
1	38.5	12.5	24.1	22875		
2	39	125	26.4	8191.7		
3	36.4	12.5	20.9	807500		
4	37.7	12.5	17.9	3400000		
5	26.9	14483.3	29.7	1775		
6	36.1	25	21.1	2400000		
7	33.7	12.5	23.4	126000		
8	25.8	535	29.8	525		
9	32	175	20.2	1209167		
10	19.5	458333.3	30.2	437.5		
11	35.4	12.5				
12	35.5	12.5				
13	34.4	25				
14	34.8	12.5				
15	31.7	100				
16	30.7	575				
17	30.3	1050				
18	29.3	225			27.4	925
19	28.4	300				
20	27.9	125				
21	22.1	136416.7				
22			36.3	25		
23			34.4	12.5		
24			33.0	25		
25			32.8	200		
26			32.0	12.5		
27			30.4	450		
28			26.9	77500		
29			22.9	57500		
30			21.9	932500		
31			19.1	560000		
32					26.8	1425

Table S1: Viral DNA (Cq values) and load of infectious virus (pfu/ml) in clinical samples.

Forty-three swabs were obtained from 32 male patients. Ten patients have both oropharyngeal and lesion swabs. One patient has both oropharyngeal and rectal swabs. LOD – limit of detection = 25 pfu/ml. Samples below LOD were assigned a value of half LOD (12.5).

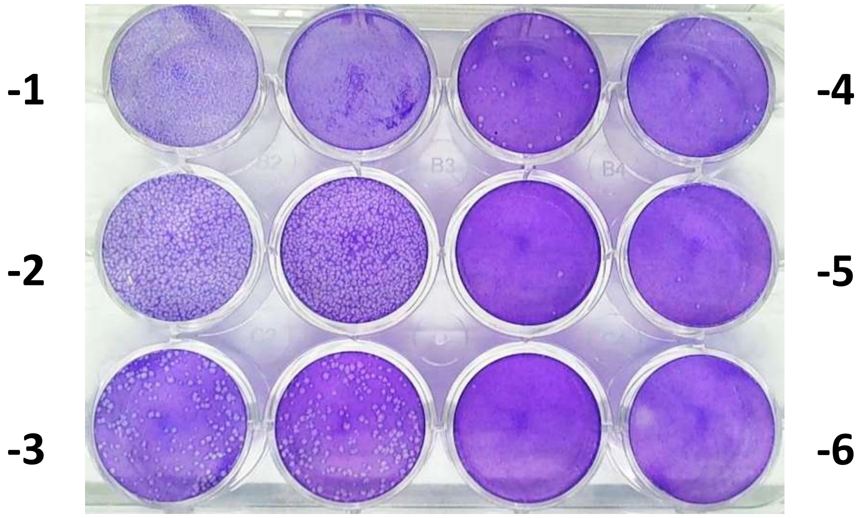

Fig. S1: Plaque forming unit (pfu) assay for MPXV clinical samples. Tenfold serial dilutions (-1 to -6) from each specimen were performed. Monolayers of BSC-1 cells in 12-well plates were infected in duplicates, incubated for 1 h at 37°C with 5% CO_2 incubator. Then 2ml of methylcellulose overlay were added to each well and cells were incubated for 72 h at 37°C with 5% CO_2 incubator. Plates were fixed and stained with crystal violet. Representative plate is shown.

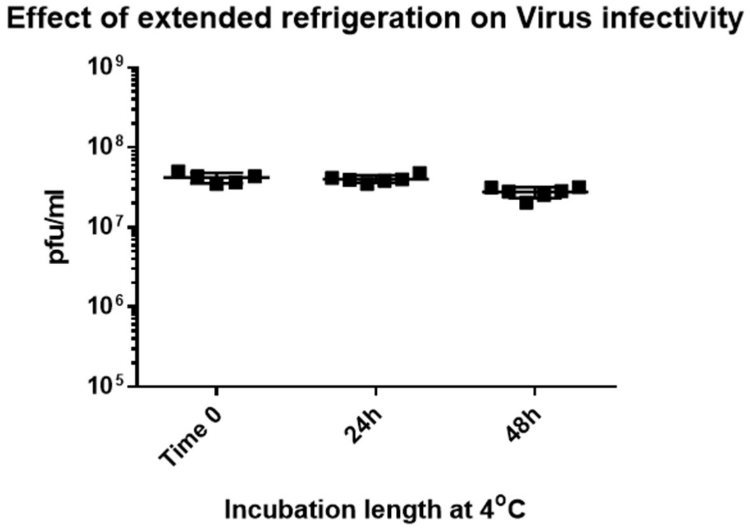

Fig. S2: Effect of refrigeration on Orthopox virus infectivity

Vaccinia Virus (Lister) purified from cell culture were stored in aliquots for up to 48 hours at 4°C and titrated on Vero cells by plaque assay.

Supplementary References:

1. Israely T, Paran N, Erez E, Cherry-Mimran L, Tamir H, Achdout H, Politi B, Israeli O, Zaide G, Cohen-Gihon I, Vitner E.B, Lustig S, and Melamed S. Differential Response Following Infection of Mouse CNS with Virulent and Attenuated Vaccinia Virus Strains. Vaccines 2019, 7, 19; doi:10.3390/vaccines7010019.
2. Li Y, Zhao H, Wilkins K, Hughes C, Damon IK. Real-time PCR assays for the specific detection of monkeypox virus West African and Congo Basin strain DNA. J Virol Methods. 2010 Oct;169(1):223-7. doi: 10.1016/j.jviromet.2010.07.012. Epub 2010 Jul 17. PMI: 20643162.
3. Rossmanith P, Wagner M. A novel poisson distribution-based approach for testing boundaries of real-time PCR assays for food pathogen quantification. J Food Prot. 2011 Sep;74(9):1404-12. doi: 10.4315/0362-028X.JFP-10-458. PMID: 21902908.
